# Synergistic Integration of Polypyrrole, Graphene Oxide, and Silver Nanowires into Flexible Polymeric Films for EMI Shielding Applications

**DOI:** 10.3390/molecules30214221

**Published:** 2025-10-29

**Authors:** Brankica Gajić, Marija Radoičić, Muhammad Yasir, Warda Saeed, Silvester Bolka, Blaž Nardin, Jelena Potočnik, Danica Bajuk-Bogdanović, Gordana Ćirić-Marjanović, Zoran Šaponjić, Svetlana Jovanović

**Affiliations:** 1 “Vinča” Institute of Nuclear Sciences, National Institute of Republic of Serbia, University of Belgrade, Mike Petovića Alasa 12-14, 11000 Belgrade, Serbia; brankica.gajic@vin.bg.ac.rs (B.G.); jpotocnik@vin.bg.ac.rs (J.P.); svetlanajovanovic@vin.bg.ac.rs (S.J.); 2 Division of Microrobotics and Control Engineering, Department of Computing Science, Carl von Ossietzky Universität Oldenburg, 26129 Oldenburg, Germany; muhammad.yasir@uni-oldenburg.de (M.Y.); warda.saeed@uni-oldenburg.de (W.S.); 3 Faculty of Polymer Technology, Ozare 19, 2380 Slovenj Gradec, Slovenia; silvester.bolka@ftpo.eu (S.B.); blaz.nardin@ftpo.eu (B.N.); 4 Faculty of Physical Chemistry, University of Belgrade, Studentski Trg 12-16, 11158 Belgrade, Serbia; danabb@ffh.bg.ac.rs (D.B.-B.); gordana@ffh.bg.ac.rs (G.Ć.-M.); 5 Institute of General and Physical Chemistry, Studentski Trg 12-16, 11158 Belgrade, Serbia; zsaponjic@iofh.bg.ac.rs

**Keywords:** polypyrrole, silver nanowires, graphene oxide, electromagnetic interference shielding, composites

## Abstract

The remarkable growth of high-frequency electronic systems has raised concerns about electromagnetic interference (EMI), emphasizing the need for lightweight and efficient shielding materials. In this study, ternary composites based on polypyrrole (PPy), graphene oxide (GO), and silver nanowires (AgNWs) were synthesized through chemical oxidative polymerization of pyrrole monomer and embedded into polycaprolactone (PCL) matrices to create flexible films. Structural and morphological analyses confirmed the successful incorporation of all components, with scanning electron microscopy showing granular PPy, sheet-like GO, and fibrous AgNWs, while spectroscopic studies indicated strong interfacial interactions without damaging the PPy backbone. Thermomechanical analysis revealed that GO increased stiffness and defined the glass transition, whereas AgNWs improved toughness and energy dissipation; their combined use resulted in balanced properties. EMI shielding effectiveness (SE) was tested in the X-band (8–12 GHz). Pure PPy exhibited poor shielding ability, while the addition of GO and AgNWs significantly enhanced performance. The highest EMI SE values were observed in PPy/GO–AgNWs composites, with an average SE of 16.05 dB at 20 wt% of the composite in the PCL matrix, equivalent to about 84.4% attenuation of incident waves. These results demonstrate that the synergistic integration of GO and AgNWs into PPy matrices enables the creation of lightweight, flexible films with advanced EMI shielding properties, showing great potential for next-generation electronic and aerospace applications.

## 1. Introduction

The rapid growth in the use of high-frequency electronic systems—ranging from wireless communication devices and smart robotics to aerospace instruments and wearable electronics—has made electromagnetic interference (EMI) an increasingly important issue. EMI, caused by the unintentional emission of electromagnetic radiation by electronic components, not only disrupts sensitive nearby devices but also potentially harms human health. With the development of compact, multifunctional, and densely integrated electronics, creating efficient, lightweight, and environmentally durable shielding materials has become crucial in ensuring electromagnetic compatibility and protecting both equipment and users [1]. Traditionally, metals such as copper, aluminum, and silver have been used as standard EMI shielding materials because of their high electrical conductivity and reflectivity. However, these conventional materials are often heavy, rigid, prone to corrosion, and unsuitable for flexible or miniaturized devices. Additionally, metallic shielding mainly relies on reflection, which can lead to secondary electromagnetic pollution, making absorption-based shielding more desirable in modern technology [2,3]. Recently, conducting polymers have emerged as promising materials for EMI shielding due to their low density, tunable electrical conductivity, chemical stability, and ease of processing. Among these, PPy has attracted significant interest because of its excellent intrinsic conductivity, environmental durability, and ability to form various micro- and nanostructures that help attenuate waves through dipole polarization and conduction losses [4]. However, pure PPy’s EMI shielding effectiveness is often limited by its brittleness, high conductivity, and poor interfacial compatibility within composite matrices [5]. To overcome these issues, hybrid composite strategies incorporating carbon- based nanomaterials and metal nanoparticles have proven highly effective [6,7]. Graphene oxide (GO), for example, offers a large specific surface area, functional surface chemistry, and high electrical conductivity (upon partial or full reduction), making it ideal for enhancing interfacial polarization and electrical pathways in the polymer matrix [8,9]. In parallel, incorporating silver nanostructures—such as silver nanowires (AgNWs) or nanoparticles (AgNPs)—into conducting polymer matrices has been shown to significantly improve electrical conductivity and EMI SE by creating additional conductive networks and promoting surface plasmon resonance effects [9,10]. These metallic inclusions contribute not only through reflection and absorption but also facilitate Joule heating and eddy current losses, further enhancing overall shielding performance [11]. Yu et al. studied conductive composites containing silver nanowires (AgNWs) and silver nanoparticles (AgNPs), demonstrating that Ag nanostructures offer superior electrical conductivity and form more efficient conductive pathways within the polymer matrix, resulting in higher EMI shielding effectiveness compared to bulk metallic fillers [12].

Previous studies have extensively demonstrated the potential of conducting polymers and hybrid nanostructures for EMI shielding. For instance, PPy-based nanostructures embedded in a silicone matrix show notable EMI shielding ability, emphasizing the importance of morphology and electrical conductivity in the shielding mechanism [13]. Composites made with GO and AgNWs reveal that combining 2D GO and 1D AgNWs improves charge transport and overall shielding efficiency [14]. GO-functionalized carbon-fiber/cement composites demonstrate better dispersion and interfacial contact, leading to a significant increase in shielding effectiveness compared to pure systems [15]. AgNW-coated textiles maintain high flexibility and light weight while providing effective EMI shielding [16]. Additionally, AgNWs/PPy hybrid systems are effective in shielding due to the synergistic interaction between metallic and conjugated polymer phases [17]. The combined integration of PPy, GO, and Ag nanostructures within a single composite can create multifunctional materials that are lightweight, flexible, and offer high EMI attenuation, wide absorption bandwidths, and environmental stability. These ternary nanocomposites utilize multiple shielding mechanisms—including dielectric polarization, interfacial and internal reflections, and better impedance matching—making them suitable for next-generation electromagnetic shielding, especially in the X- and Ku-bands. [18,19].

Although many studies focus on conducting polymer-based composites for EMI shielding, most reported systems still face issues like poor flexibility, limited processability, and discontinuous conductive networks, especially when using free-standing films or rigid matrices. Furthermore, while binary PPy/GO and PPy/AgNWs composites have demonstrated promising electrical properties, their combination in a flexible polymer matrix like polycaprolactone (PCL) has not been thoroughly investigated. The synergistic integration of PPy, GO, and AgNWs in a PCL matrix aims to solve these problems by combining the high electrical conductivity of AgNWs, the large surface area and interfacial polarization of GO, and the environmental stability of PPy within a flexible, processable host polymer. This method creates a new pathway toward lightweight, mechanically robust, and highly effective EMI shielding materials suitable for flexible electronics and environmental protection.

To the best of our knowledge, this study reports for the first time the synthesis of flexible and processable films based on polypyrrole (PPy), graphene oxide (GO), and silver nanowires (AgNWs), embedded in a polycaprolactone (PCL) matrix, demonstrating promising electromagnetic interference (EMI) shielding capabilities.

## 2. Results and Discussion

### 2.1. Morphological Properties of Synthesized Materials

**Figure 1:**
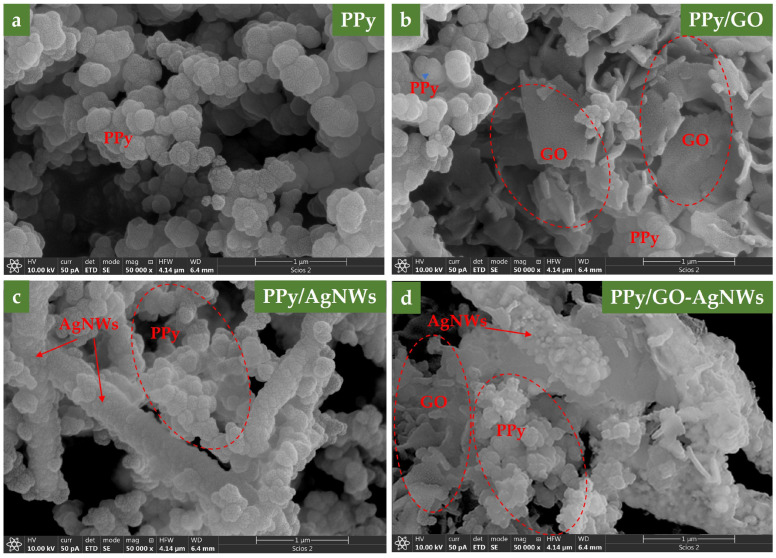
SEM micrographs of neat PPy (**a**), PPy/GO (**b**), PPy–AgNWs (**c**), and PPy/GO–AgNWs (**d**).

**Figure 2:**
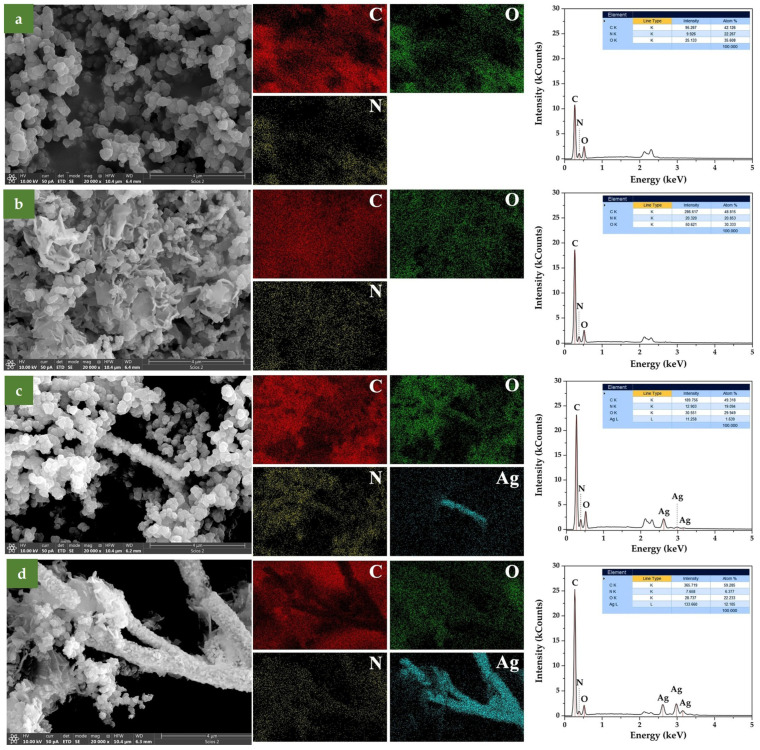
EDS elemental mapping and spectra for (**a**) PPy, (**b**) PPy/GO, (**c**) PPy/AgNWs, and (**d**) PPy/GO–AgNWs. Elemental distribution is shown using different colors: red for carbon, green for oxygen, yellow for nitrogen, and cyan for silver.

SEM micrographs of all synthesized samples are shown in Figure 1. To verify the chemical composition of the examined samples, elemental mapping was performed using EDS. The results are presented in Figure 2, along with the corresponding spectra, shown in the 0 to 5 keV energy range for better clarity. In the elemental maps, different colors were assigned to each element to emphasize their spatial distribution: carbon (C) in red, oxygen (O) in green, nitrogen (N) in yellow, and silver (Ag) in cyan.

Regarding polymerization under acidic conditions, the pure PPy sample (Figure 1a) exhibited typical granular morphology, with an average particle size of about 150 nm [20]. The granular particle size and distribution are shown in SI, Figure S1. The original sizes of the pure GO and AgNWs samples before in situ polymerization of pyrrole are available from our previous study by Milenković et al. [19]. Adding GO dispersion to the polymerization mixture resulted in a mixed morphology PPy/GO composite that displayed features of both components, such as a two-dimensional sheet-like graphene structure and granular-shaped PPy. SEM images of the PPy/GO composite reveal that the GO sheet’s lateral size ranges from a few hundred nanometers to several micrometers (Figure 1b). After polymerization, some differences from the original dimensions are noticeable, caused by the formation of a polypyrrole layer on the GO surface, confirmed by the presence of N atoms in elemental mapping (Figure 2b). In the pure GO sample, only C and O atoms were detected, with no N atoms present, as expected (SI, Figure S2).

When pyrrole polymerization was carried out in the presence of AgNWs, the resulting composite materials displayed silver nanowires coated with a granular PPy layer (Figure 1c). The PPy-coated nanowires are over 4 μm long, with an average diameter of about 600 nm, depending on the thickness of the PPy layer on the surface of the AgNWs. The detection of nitrogen in the elemental mapping of GO and AgNWs clearly confirms the successful deposition of the polypyrrole layer on their surfaces.

Furthermore, after synthesizing the hybrid PPy/GO–AgNWs system, the distinct morphological features of all three components are clearly visible, demonstrating that each component maintained its original structure while being evenly coated with a PPy layer (Figure 1d). Elemental mapping also confirmed the presence of nitrogen on both GO and AgNWs surfaces, providing direct evidence that these components are covered with a polypyrrole layer (Figure 2d).

Besides microscopy detection, AgNWs are clearly visible in elemental mapping (Figure 2c,d), confirming their presence in the PPy/AgNWs and PPy/GO–AgNWs samples.

The EDS spectra from the examined areas display characteristic peaks at 0.27 keV, 0.38 keV, and 0.52 keV, corresponding to the K lines of carbon, nitrogen, and oxygen, respectively. The presence of silver is clearly identified by its L lines at 2.62 keV, 2.98 keV, and 3.15 keV (Figure 2c,d). Additionally, peaks near 2.1 keV and 2.3 keV originate from the M lines of gold, which was used during sample preparation for SEM imaging.

EDS elemental mapping clearly revealed a uniform distribution of nitrogen across all composite samples, confirming the successful deposition of a PPy layer onto their surfaces (PPy/GO, PPy/AgNWs, PPy/GO–AgNWs). The minor variations in elemental composition likely stem from local morphological differences. Since polypyrrole is the only nitrogen-containing component, these results strongly support the formation of a continuous PPy coating on the surfaces of GO and AgNWs, explaining the dimensional differences observed compared to the initial precursors.

### 2.2. Molecular Structure

**Figure 3:**
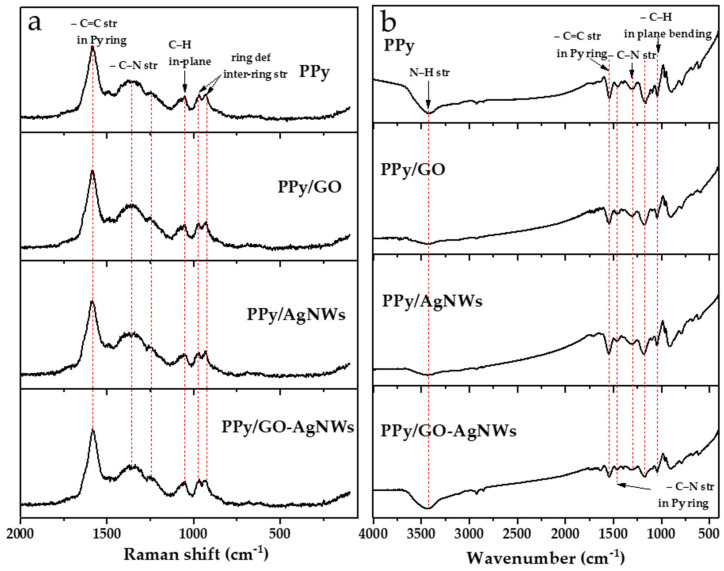
Raman (**a**) and FTIR (**b**) spectra of synthesized neat PPy and PPy-based composite materials.

The molecular structure of the synthesized materials was analyzed using Raman and FTIR spectroscopies (Figure 3).

The Raman spectra of all composites based on neat PPy and all PPy-based nanocomposites (PPy/GO, PPy/AgNWs, and PPy/GO–AgNWs) are shown in Figure 3a. The most intense band in all spectra, observed at 1588 cm^−1^, corresponds to the stretching vibrations of C=C bonds within the polymer backbone, indicating the presence of a π-conjugated system in PPy [21]. The band at 1370 cm^−1^ is attributed to stretching vibrations within the pyrrole ring, while the band at 1248 cm^−1^ is associated with the stretching of bonds connecting individual pyrrole units along the polymer chain, confirming the successful formation of the polymer structure [22,23,24]. The band at 1048 cm^−1^ may relate to C–H bending or in-ring deformation modes [23]. Particularly prominent bands at 968 and 930 cm^−1^ are assigned to ring deformations caused by charged species, specifically polarons and bipolarons, further confirming the conductive nature of the obtained PPy.

Although GO is present in the composite structure, the Raman spectra of the PPy/GO composite closely resemble that of pure PPy, without clearly visible D and G bands typically associated with GO (D band at ~1350 cm^−1^ and G band between 1580 and 1600 cm^−1^) [25]. This suggests that GO is likely not just physically mixed but is instead incorporated and well dispersed within the polymer matrix, so that PPy chains largely cover GO sheets [26]. Strong interactions between the PPy chains and the functional groups on the GO surface, as well as the lower GO content relative to PPy, may cause the GO bands to be masked or less distinguishable in the composite spectrum.

AgNWs do not show their own characteristic Raman bands and are mainly used as substrates to enhance signals in SERS (Surface-Enhanced Raman Scattering) systems [27]. If the polymer matrix completely covers the AgNWs or if they are not exposed on the surface, their contribution to the Raman spectrum is minimal. This may explain why there are no significant differences between the spectrum of pure PPy and that of the PPy/AgNWs composite [28]. Due to the reasons discussed for binary composites, the Raman spectrum of the ternary composite PPy/GO–AgNWs is also dominated by PPy bands and is very similar to the spectra of PPy, PPy/GO, and PPy/AgNWs.

FTIR spectra of all synthesized materials are presented in Figure 3b. The spectra of pure PPy, along with PPy/GO, PPy/AgNWs, and PPy/GO–AgNWs composites, show characteristic vibrational bands of PPy, confirming that the polymer’s core chemical structure remains intact [29]. This indicates that the synthesis of the composites did not cause significant chemical changes in the PPy component.

In all spectra, the following bands typical of PPy were observed. The broad band at 3425 cm^−1^ is attributed to N–H stretching vibrations, while the bands at 1540 cm^−1^ and 1450 cm^−1^ correspond to C–C and C–N stretching vibrations in the pyrrole ring, respectively [21,22,30,31]. The band at approximately 1300 cm^−1^ is associated with in-plane deformation vibrations of the C–N and C–H groups, whereas the band at 1167 cm^−1^ corresponds to the pyrrole ring “breathing” vibrations [21,32]. The band at 1038 cm^−1^ can be assigned to C–H and N–H deformation vibrations, while a weaker band at 888 cm^−1^ relates to out-of-plane C–H vibrations characteristic of the pyrrole ring [22,29].

In the spectra of the PPy/GO and PPy/GO–AgNWs composites, the bands originating from GO (C=O stretching at ~1735 cm^−1^ and epoxy C–O–C stretching at ~1087 cm^−1^) are not clearly visible. The strong broad band at 3425 cm^−1^, due to N-H stretching in PPy [25,33], most likely overlaps with the GO band from O-H stretching, which appears at a nearby position of ~3440 cm^−1^ [34]. This can be explained by the fact that PPy coats the composite surface and dominates the IR absorption, while GO and AgNWs are mostly embedded in the matrix and present in smaller amounts, causing their signals to be masked by the polymer’s bands. In the spectra of the composites, the band caused by N-H stretching vibration is at nearly the same position as in pristine PPy (at 3425 cm^−1^). Differences in its shape and relative intensity among the spectra of the synthesized materials could result from various factors, such as changes in hydrogen bonding interactions (e.g., replacement of N-H····N-H in pure PPy with N-H····O-H in composites due to the presence of GO) or different amounts of residual adsorbed water, which depend on their surface properties like hydrophilicity. These spectroscopy results confirm the successful incorporation of GO and AgNWs into the PPy matrix while maintaining the polymer’s core structure.

### 2.3. Thermogravimetric Analysis

**Figure 4:**
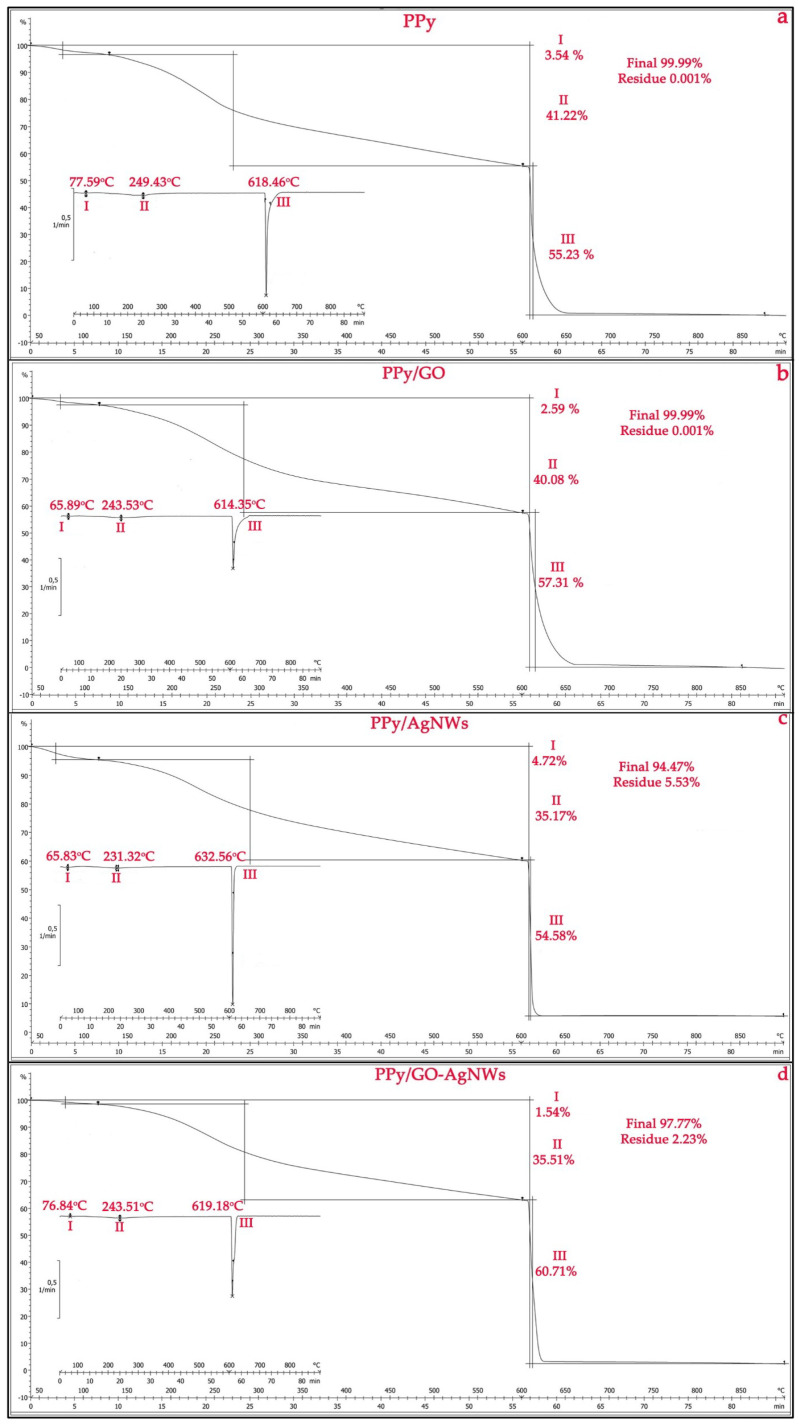
Thermogravimetric analysis of synthesized powder samples: PPy (**a**), PPy/GO (**b**), PPy/AgNWs (**c**), and PPy/GO–AgNWs (**d**).

Figure 4 shows the TGA results of pristine PPy, its composite PPy/GO, as well as the PPy/AgNWs and PPy/GO–AgNWs nanocomposites. All tested materials exhibit distinct mass loss behaviors. The initial weight loss, between 65 and 78 °C, for all samples is caused by the removal of adsorbed water from the surface [28,35]. During this stage, the mass loss of the PPy/GO composite (Figure 4b,c) is lower than that of neat PPy (Figure 4a), confirming the formation of a PPy layer on the GO sheets and AgNWs, along with a slight improvement in thermal stability during the first degradation stage [36,37]. A minor deviation is observed for the PPy/AgNWs sample, where the degradation temperature is lower than that of pure PPy, while the mass loss in the first degradation stage is slightly higher due to the presence of AgNWs. The minimal mass loss of 1.54% observed for the PPy/GO–AgNWs composite (Figure 4d) is attributed to a synergistic effect between the GO sheets and AgNWs [18], which, to some extent, alters the thermal stability of the PPy matrix. This effect results from improved heat distribution and increased mechanical strength, which slow down the thermal degradation of the polymer [38].

The thermogram of pristine PPy (Figure 4a) shows that the polymer powder loses 41.22% of its mass in N_2_ atmosphere during the second degradation step (differentiation maximum at 249.43 °C) and 55.23% during the third degradation step (differentiation maximum at 618.46 °C). A total mass loss of approximately 99.99% occurs above 620 °C in O_2_ atmosphere, indicating complete polymer decomposition [39]. A similar thermal behavior is observed for the PPy/GO composite (Figure 4b), with mass losses in similar temperature ranges: 40.08% (max 243.53 °C) during the second and 57.31% (max 614.35 °C) during the first degradation step. This demonstrates that adding GO does not change the overall degradation pattern but may slightly influence the sample’s thermal stability.

Results from the TGA analysis of the PPy/AgNWs and PPy/GO–AgNWs composites (Figure 3) show their enhanced thermal stability during the third degradation stage compared to pure PPy. Specifically, during this phase, the weight loss for the PPy/AgNWs sample is 54.58% at a maximum temperature of 632.56 °C, while the ternary PPy/GO–AgNWs nanocomposites experience a 60.71% loss at a maximum of 619.18 °C.

The final decomposition showed total mass losses of 94.47% for PPy/AgNWs and 97.77% for PPy/GO–AgNWs. The remaining masses were 5.53% for the PPy/AgNWs composite and 2.23% for the PPy/GO–AgNWs hybrid, which are due to the presence of thermally stable silver nanowires. The slight increase in thermal stability results from a synergistic interaction between the polymer and the AgNWs and GO/AgNWs, which act as thermal stabilizers and only slightly slow down polymer degradation.

### 2.4. Thermomechanical Analysis

**Figure 5:**
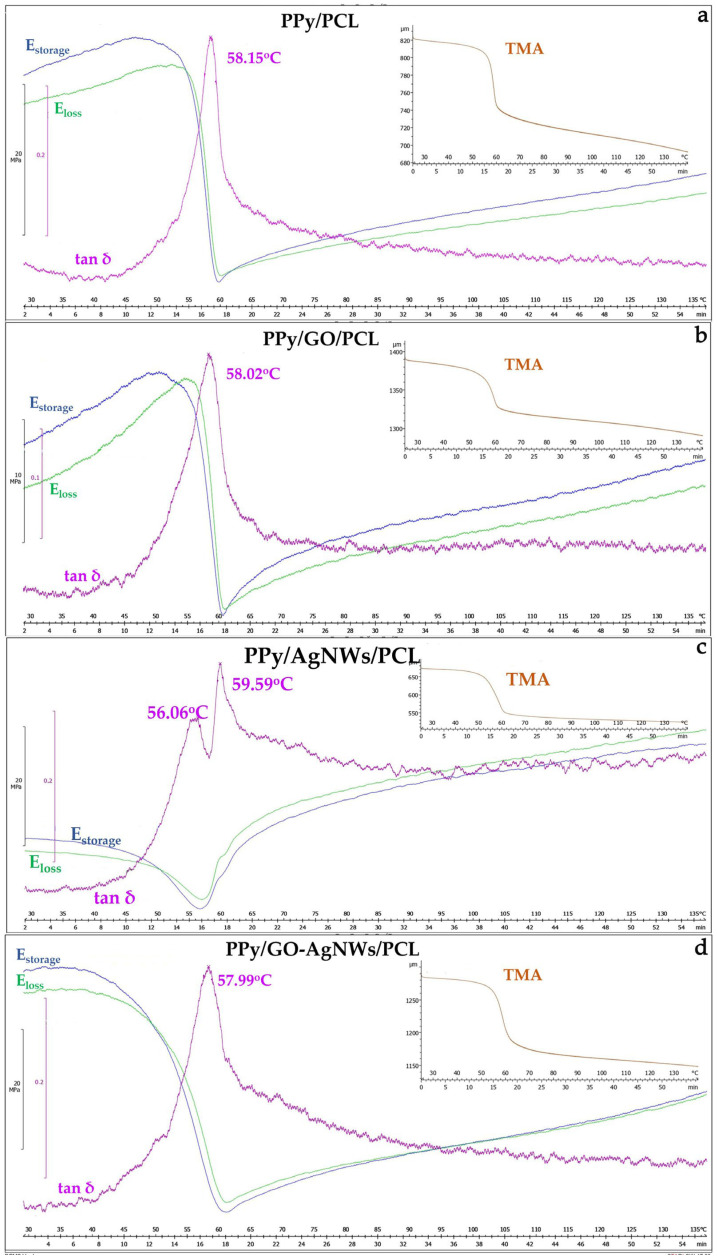
Thermomechanical analysis of synthesized film samples: PPy (**a**), PPy/GO (**b**), PPy/AgNWs (**c**), and PPy/GO–AgNWs (**d**).

Results of the TMA for all synthesized PCL film samples are shown in Figure 5. The filler content in the PCL matrix was maintained at 20 wt% for each sample. The tan δ maxima for neat PPy/PCL (58.15 °C, Figure 5a) and PPy/GO/PCL (58.02 °C, Figure 5b) were nearly identical, indicating that adding GO did not significantly alter the segmental mobility of the PCL matrix.

However, GO incorporation produced a sharper tan δ peak and higher E′ before Tg, indicating increased stiffness and effective stress transfer due to strong filler–matrix interactions. Adding sheet-like fillers such as GO into the PCL matrix enhances stiffness and stress transfer, consistent with previous findings where starch effectively reinforced PCL and improved its damping capacity for energy dissipation [40].

In contrast, PPy/AgNWs/PCL (Figure 5c) exhibited two distinct tan δ maxima (56.06 °C and 59.59 °C), indicating the presence of domains with different chain mobilities, likely caused by partial phase separation and varying filler–matrix compatibility. This phase separation between PPy and PCL domains has been confirmed in earlier studies through DSC and DMA analyses of PPy-g-PCL copolymers [41].

The PPy/GO–AgNWs/PCL (Figure 5d) hybrid exhibited a similar temperature value (57.99 °C) but with a slightly wider tan δ peak, indicating a range of local values caused by heterogeneous interfacial environments. According to the literature, adding polypyrrole into PCL-based blends has been shown to cause strong interactions with the host matrix, leading to conductivity changes and altered tensile behavior, especially in hydrated conditions [42,43]. The PPy/GO/PCL sample displayed a sharp, high-intensity tan δ peak (~1.12), consistent with a homogeneous amorphous transition. Neat PPy/PCL had a similar peak height (~1.20) but lacked the additional structural stiffening provided by GO. The AgNWs-based composite showed two distinct peaks (~1.16 and ~1.09), reflecting its strong heterophase nature. The PPy/GO–AgNWs/PCL composite exhibited a broader peak (~1.20), confirming increased microstructural heterogeneity.

The neat PPy/PCL composite had lower stiffness, as expected for a system without reinforcing nanofillers. It exhibited the lowest initial stiffness and higher E″ values over a wider temperature range, indicating improved energy dissipation and decreased structural uniformity. PPy/GO/PCL showed the highest E′ in the glassy region and a steep modulus drop at Tg, confirming a well-defined transition. The hybrid PPy/GO–AgNWs/PCL system maintained stiffness similar to PPy/GO/PCL, with a slightly less pronounced modulus drop, likely due to the synergistic reinforcing effects of the sheet-shaped (GO) and fibrous (AgNWs) fillers.

In TMA curves, neat PPy/PCL shows a sharp dimensional change at Tg, coinciding with a sudden drop in modulus. PPy/GO/PCL and the PPy/AgNWs/PCL hybrid exhibit a clear dimensional contraction near Tg, followed by linear expansion, indicating thermal stability at higher temperatures. The PPy/GO–AgNWs/PCL sample demonstrates a more gradual dimensional change, reflecting greater flexibility and a less distinct transition.

The results show that GO is the most effective at increasing stiffness and outlining the glass transition in PCL/PPy composites, while the PPy/GO–AgNWs/PCL hybrid offers a good balance of mechanical strength and potential electrical conductivity. AgNWs alone enhance toughness and flexibility but decrease stiffness and uniformity. These differences highlight the importance of filler shape and interfacial interactions in customizing the thermo-mechanical properties of conductive polymer composites.

### 2.5. EMI Shielding and Conductivity Measurements

The shielding effectiveness of flexible composite films was determined using Equations (1)–(3) [44,45]:

(1) $$SE=L_{D}+L_{M}$$

where SE of samples is calculated as the sum of dissipation loss, *L_D_*, and mismatch loss, *L_M_*_._

The value of *L_M_* is calculated using Equation (2):

(2) $$L_{M}=-10\;\log_{10}\left(1-{\left|S_{11}\right|}^{2}\right)$$

L_D_ was determined by considering the reflection scattering parameter, *S*_11_, and the transmission scattering parameter, *S*_21_, using Equation (3):

(3) $$L_{D}=-10\;\log_{10}\left({\left|S_{21}\right|}^{2}/\left(1-{\left|S_{11}\right|}^{2}\right)\right)$$

Both *S*_11_ and *S*_21_ were measured using a VNA.

**Figure 6:**
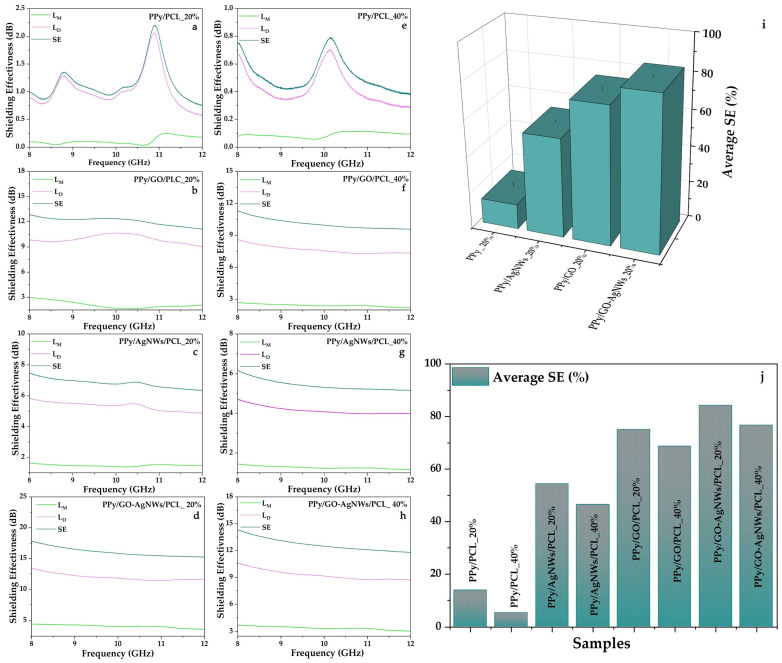
Shielding effectiveness of all prepared flexible PCL film samples at two different concentrations (20 and 40 mass%) (**a**–**h**); schematic illustration of the influence of individual components on SE (**i**), as well as the effect of composite loading on average SE value (**j**).

All prepared samples demonstrated the ability to attenuate electromagnetic waves (EMWs) within the 8–12 GHz frequency range. The effects of individual components on the EMI shielding properties of hybrid films at 20 wt% PPy-based composite (filler) loading, and the influence of filler content (20 wt% and 40 wt%) on the EMI shielding performance of the hybrid films, are shown in Figure 6. For each sample, the filler content in the PCL matrix was maintained at 20 and 40 wt%. Pristine PPy-based films with 20% (PPy/PCL_20%, Figure 6a) and 40% (PPy/PCL_40%, Figure 6e) filler loadings showed relatively low shielding effectiveness (SE), with average values of 1.15 dB and 0.47 dB, respectively. The total SE was mainly due to dissipation loss, while mismatch loss was negligible. This behaviour aligns with previous reports on pure PPy films, which generally display limited EMI shielding performance because of their modest conductivity and poor processability [4].

Further enhancement was achieved by incorporating GO into PPy. The average SE values increased to 12.07 dB and 10.08 dB for PPy/GO/PCL_20% (Figure 6b) and PPy/GO/PCL_40% (Figure 6f), respectively. As with previous cases, dissipation loss remained the primary shielding mechanism. This improvement can be attributed to interfacial polarization between GO sheets and the PPy matrix, which enhances multiple scattering and absorption. Similar findings have been reported for graphene/PPy composites, where constructing conductive networks significantly improved EMI shielding effectiveness [4].

Adding AgNWs to the PPy matrix significantly enhanced the EMI response. The average SE recorded was 6.81 dB for PPy/AgNWs/PCL_20% (Figure 6c) and 5.42 dB for PPy/AgNWs/PCL_40% (Figure 6g) samples. In both cases, dissipation loss (*L_D_*) was the main factor, while mismatch loss (*L_M_*) remained low. Similar improvement trends have been observed in silane-modified PPy–Ag nanocomposite films, where silver nanoparticles increased conductivity, resulting in a 32% increase in EMI SE across a wide frequency range.

The highest performance was observed in quaternary PPy/GO–AgNWs/PCL composites. The PPy/GO–AgNWs/PCL_20% (Figure 6d) sample demonstrated the most effective shielding, blocking about 84.4% of incoming EM radiation with an average SE of 16.05 dB. The 40% composite (PPy/GO–AgNWs/PCL_40%, Figure 6h) achieved a slightly lower SE of 12.66 dB. While dissipation loss was the primary shielding mechanism, mismatch loss was more evident in the binary composite, indicating improved impedance matching due to the synergistic effect of AgNWs and GO. This finding aligns with recent studies on PPy/Ag/graphene ternary composites, which reported SET values over 30 dB in the Ku-band, primarily driven by absorption mechanisms [11]. A detailed study on the interaction between AgNWs and GO was provided by Milenković et al. [19].

The 3D representation (Figure 6i) clearly visualises the progressive enhancement in SE, whereas the comparative bar chart (Figure 6j) highlights the combined effects of filler composition and loading content (20 wt% and 40 wt%) on the overall SE performance. The experimental results demonstrated an apparent increase in EMI shielding efficiency, progressing from pristine PPy/PCL to ternary PPy/GO/PCL or PPy/AgNWs/PCL, and finally to quaternary PPy/GO–AgNWs/PCL. This trend can be attributed to the gradual enhancement of charge transport pathways, increased interfacial polarization, and the development of a more continuous and effective conductive network within the polymer matrix [13]. Similar hierarchical improvements have been observed in polymer nanocomposites reinforced with carbon nanotubes [46], PPy/carbon nanofiber hybrid films [47], and graphene/multiwalled carbon nanotube hybrids [48]. Overall, these findings confirm that customizing the composite structure is essential for creating high-performance, lightweight, and absorption-focused EMI shielding materials.

The observed EMI shielding performance of the synthesized PPy/GO–AgNWs composites can be compared to previously reported systems based on conductive polymers and hybrid nanostructures. For example, Moučka et al. [13] studied PPy nanostructures with various morphologies (globules, nanotubes, and microbarrels) embedded in a silicone matrix. They demonstrated their EMI shielding ability in the C-band range (5.85–8.2 GHz). They showed that morphology and electrical conductivity influence the shielding mechanism. Sim et al. [14] developed graphene GO/AgNW composites with excellent EMI SE (up to 92 dB at 8.2 GHz) and even self-healing properties, illustrating that highly conductive hybrid 2D/1D architectures can deliver superior performance. Chen et al. [15] reported GO-deposited carbon-fiber cement composites with improved dispersion and interfacial contact, resulting in a 31% increase in SE compared to pure CF/cement due to GO functionalization. Liu et al. [16] prepared AgNW-modified textiles that achieved a 59 dB EMI SE in the 5–18 GHz range, highlighting their flexibility and low density. Moreover, Wang et al. [17] created PPy/PDA/AgNW composites with adjustable electrical conductivity (0.01–1206 S/cm) and EMI SE reaching up to 48 dB in the X-band, demonstrating the advantage of AgNWs in developing efficient conductive networks within the PPy matrix.

The conductivity measurements follow entirely the trend seen in the SE results. The lowest conductivity was found in the pure PPy sample, with a value of 3.84 S m^−1^. Conductivity gradually rises, reaching 4.7 S m^−1^ for the PPy/GO sample and 6.1 S m^−1^ for PPy/AgNWs. The highest conductivity (8.2 S m^−1^) was recorded for the sample showing the highest SE value. These findings further support the SE analysis and emphasize the strong link between electrical conductivity and EMI shielding performance.

## 3. Materials and Methods

### 3.1. Materials

Pyrrole (Py, p.a. 98%, Sigma-Aldrich, St. Louis, MO, USA), ammonium peroxydisulfate (APS, T.T.T. d.o.o., Sveta Nedelja, Croatia), polycaprolactone (PCL, Thermo Scientific, NJ, USA), hydrochloric acid (HCl, 32%, Fluka Chemie GmbH, Buchs, Switzerland), ethanol (C_2_H_5_OH, ≥99.8%, Fisher Scientific, Loughborough, UK), chloroform (CHCl_3_, Macron Fine Chemicals™, Gliwice, Poland), and deionized water were used as received, without further purification.

### 3.2. Synthesis of PPy/GO, PPy/AgNWs and PPy/GO/AgNWs Composites and Their Appropriate Films in PLC

Before preparing the composite, graphene oxide (GO), silver nanowires (AgNWs), and their hybrid combinations (AgNWs/GO) were synthesized using the procedure described by Milenković et al. [19].

Flexible films based on PPy composites with GO, AgNWs, and their hybrid combination (GO–AgNWs), incorporated into a high-viscosity polycaprolactone solution of PCL in chloroform, were synthesized through a two-step process.

The first step involved chemical oxidative polymerization of Py in an acidic medium, using APS as an oxidant. Solutions of APS (20 mmol in 20 mL of 1.2 M HCl) and Py (16 mmol in 20 mL of 1.2 M HCl) were added simultaneously to previously prepared aqueous dispersions of GO, AgNWs, or their hybrid (GO/AgNWs) (100 mL, 1 mg/mL). The reaction mixture was stirred at room temperature for 1 h. The resulting precipitated nanocomposite was collected by filtration, washed with 1.2 M HCl in ethanol, and then dried under vacuum at 60 °C for 3 h. Pristine PPy was synthesized using the same procedure without adding GO, AgNWs, or GO-AgNWs hybrid.

In the second step, the synthesized nanocomposites were added to a viscous solution of PCL in chloroform to create composite films with enhanced mechanical properties. Specifically, 0.1 g of each nanocomposite was mixed into the PCL solution (9.6 g of PCL dissolved in 75 mL of chloroform). The mixtures were stirred until uniform dispersions were achieved, then cast into molds to form films. The films were left to dry at room temperature until the solvent fully evaporated.

### 3.3. Methods

Scanning electron microscopy (SEM) analysis of the PPy, PPy/GO, PPy/AgNWs, and PPy/GO–AgNWs samples was conducted using a SCIOS 2 Dual Beam scanning electron microscope from Thermo Fisher Scientific in Waltham, MA, USA, equipped with an energy dispersive X-ray spectrometer (EDS) and operated at an accelerating voltage of 10 kV. EDS elemental mapping was performed at 10 kV and a magnification of 20,000×, with an acquisition time of 30 min. Before SEM imaging, the samples were sputter-coated with gold (Au).

Raman and Fourier Transform Infrared (FTIR) spectroscopies were used to analyze the molecular structure of powder nanocomposites (PPy, PPy/GO, PPy/AgNWs, and PPy/GO–AgNWs), as well as the interactions between the individual components. For Raman spectroscopy measurements, as-synthesized powder samples were used. For FTIR measurements, as-synthesized nanocomposite powders (5 mg each) were mixed and homogenized with 100 mg KBr and pressed into pellets before measurements. Raman spectra of powder samples were collected using a DXR Raman microscope (Thermo Fisher Scientific, Waltham, MA, USA) with a 532 nm laser. For each sample, three different spots were measured. The laser power was 2 mW, and the acquisition time was set to 10 × 10 s. FTIR spectra were recorded using a Nicolet iS20 spectrometer (Thermo Scientific, Waltham, MA, USA) equipped with a diamond ATR crystal. The powder composites were placed directly on the crystal, and FTIR spectra were collected at a resolution of 4 cm^−1^ with 16 scans per spectrum.

The thermal stability of GO, AgNWs, and GO/AgNWs composites with PPy was evaluated using a TGA/DSC 3+ instrument (Mettler Toledo GmbH, Greifensee, Switzerland). About 3 mg of each sample was tested under a nitrogen atmosphere at a flow rate of 20 mL/min from 40 °C to 600 °C, then under an oxygen atmosphere at 20 mL/min from 600 °C to 900 °C. The temperature increased at a constant heating rate of 10 K/min. Each test was performed twice to confirm reproducibility.

Thermomechanical analysis (TMA) was conducted to evaluate the mechanical properties under dynamic and thermal conditions using a TMA7SDTA 2+ instrument (Mettler Toledo GmbH, Greifensee, Switzerland). The sample dimensions are 4 × 4 × 1 mm^3^ and were subjected to a heating cycle from 22 °C to 140 °C at a constant rate of 2 °C/min. The analysis was conducted under sinusoidal mechanical stress, with the force oscillating between 0.05 N and 0.5 N at a cycle period of 6 s, in a nitrogen environment (30 mL/min).

To determine the EMI SE of PPy-based composites, samples were cast into a 15 mm × 25 mm mold. The sample dimensions matched the inner size of WR-90 waveguide adapters used for electromagnetic shielding tests. For S-parameter measurements, we used a vector network analyzer (VNA, a Rohde & Schwarz ZVA 24 Vector Network Analyzer, Munich, Germany). The frequency range covered 8 GHz to 12 GHz. WR-90 waveguide adapters were connected to ports 1 and 2 of the VNA with RF coaxial cables. The thin film was placed between the two waveguide adapters, and the S21 scattering parameters were recorded for all PPy composites, following the procedure described earlier [49].

The sheet resistance of the samples was measured by the four-point probe method (JANDEL RM 3000).

**Scheme 1:**
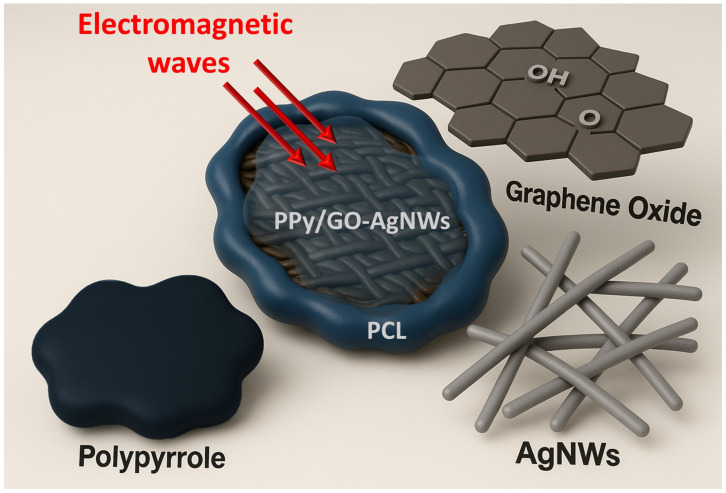
Schematic illustration of the synthesized PPy/GO–AgNWs composite for EMI shielding application.

Generative artificial intelligence (GenAI) tools were used to generate sections of Scheme 1 and assist with grammatical editing of the manuscript.

## 4. Conclusions

In this study, novel PPy-based composites reinforced with GO and AgNWs were synthesized, structurally characterized, and assessed for their thermomechanical and EMI shielding performance. SEM analysis confirmed the successful creation of PPy-based nanocomposites, showing granular PPy, sheet-like GO, and fibrous AgNWs, each maintaining their characteristic morphology within the hybrid system. Raman and FTIR spectra further verified the preservation of the polymer backbone, confirming that both fillers were incorporated without disrupting the conjugated polymer structure. TGA measurements indicated a slight improvement in the thermal stability of the hybrid PPy/GO–AgNWs composites compared to pure PPy. Before TMA and EMI shielding testing, film samples in PCL were prepared with 20 and 40 wt% loadings of the synthesized PPy-based composite. TMA revealed the supporting role of GO, which increased stiffness and defined the glass transition, while AgNWs contributed toughness, and their hybrid combination provided a balanced response.

EMI shielding tests in the X-band (8–12 GHz) showed that neat PPy/PCL had low effectiveness, while adding GO and AgNWs significantly improved performance. The highest shielding was observed in ternary PPy/GO–AgNWs/PCL_20 wt% composites, with an average SE of 16.05 dB, indicating 84.4% attenuation of incident electromagnetic waves. Dissipation loss was the primary mechanism, although mismatch loss became more significant in ternary systems.

Overall, combining GO, AgNWs, and PPy into the PCL matrix produced lightweight, flexible, and durable films suitable for advanced EMI shielding in various electronic applications.

## Data Availability

The datasets analyzed in the current study are available in the Zenodo repository (https://doi.org/10.5281/zenodo.17059983).

## Supplementary Materials

The following supporting information can be downloaded at: https://www.mdpi.com/article/10.3390/molecules30214221/s1, Figure S1. Granular particle size and its size distribution of PPy nanoparticles. Figure S2. Elemental mapping and EDS spectra of neat GO.
