# Supplementary material for: Synergistic Integration of Polypyrrole, Graphene Oxide, and Silver Nanowires into Flexible Polymeric Films for EMI Shielding Applications
Source: Molecules. 2025 Oct 29;30(21):4221. doi: 10.3390/molecules30214221 (PMC12610193; doi:10.3390/molecules30214221)
Supplement: Supplementary file 1 [file molecules-30-04221-s001.zip › molecules-3896113-supplementary.pdf]

Supplementary Material

Synergistic Integration of Polypyrrole, Graphene Oxide, and Silver Nanowires into Flexible Polymeric Films for EMI Shielding Applications

Brankica Gajić [1], Marija Radoičić [1,*], Muhammad Yasir [2], Warda Saeed [2], Silvester Bolka [3], Blaž Nardin [3], Jelena Potočnik [1], Danica Bajuk-Bogdanović [4], Gordana Ćirić-Marjanović [4], Zoran Šaponjić [5] and Svetlana Jovanović [1]

[1] "Vinča" Institute of Nuclear Sciences, National Institute of Republic of Serbia, University of Belgrade, Mike Petovića Alasa 12-14, 11000 Belgrade, Serbia; brankica.gajic@vin.bg.ac.rs (B.G.); jpotocnik@vin.bg.ac.rs (J.P.); svetlanajovanovic@vin.bg.ac.rs (S.J.)
[2] Division of Microrobotics and Control Engineering, Department of Computing Science, Carl von Ossietzky Universität Oldenburg, 26129 Oldenburg, Germany; muhammad.yasir@uni-oldenburg.de (M.Y.); warda.saeed@uni-oldenburg.de (W.S.)
[3] Faculty of Polymer Technology, Ozare 19, 2380 Slovenj Gradec, Slovenia; silvester.bolka@ftpo.eu (S.B.); blaz.nardin@ftpo.eu (B.N.)
[4] Faculty of Physical Chemistry, University of Belgrade, Studentski Trg 12-16, 11158 Belgrade, Serbia; danabb@ffh.bg.ac.rs (D.B.-B.); gordana@ffh.bg.ac.rs (G.Ć.-M.)
[5] Institute of General and Physical Chemistry, Studentski Trg 12-16, 11158 Belgrade, Serbia; zsaponjic@iofh.bg.ac.rs
* Correspondence: mradoicic@vin.bg.ac.rs

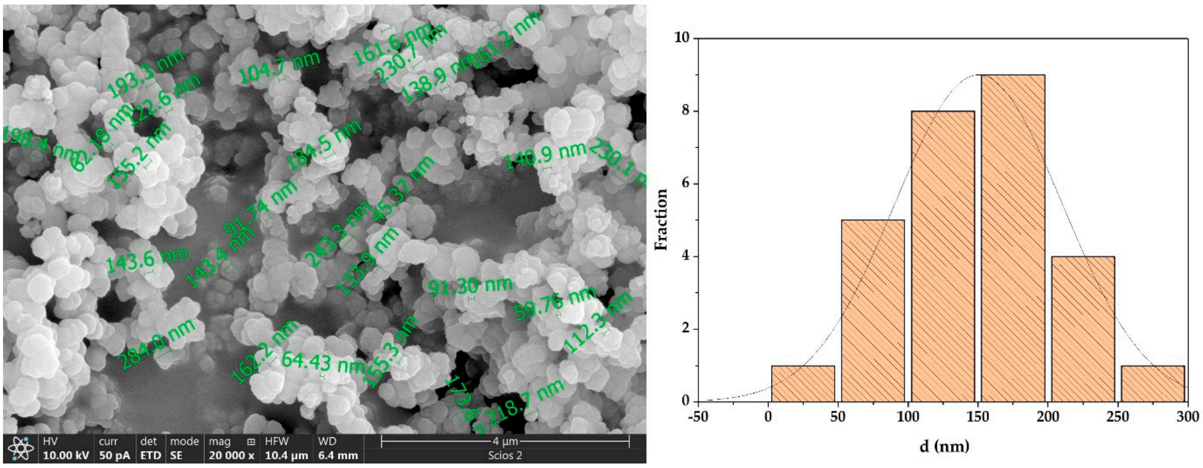

Figure S1. Granular particle size and its size distribution of PPy nanoparticles.

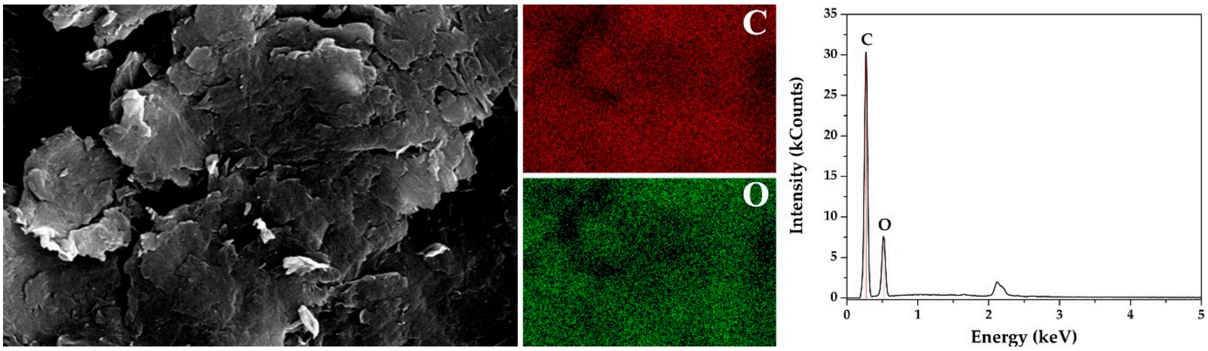

Figure S2. Elemental mapping and EDS spectra of neat GO.
